# Aneurysmal bone cyst associated with a dentigerous cyst in the mandible: A diagnostic challenge—case report

**DOI:** 10.1016/j.radcr.2025.11.021

**Published:** 2025-12-15

**Authors:** Fereshteh Hayatimotlagh, Mohammad Taghi Mashkouri Najafi, Bita Heydarzadeh, Saba Khorram

**Affiliations:** a Department of Oral and Maxillofacial Radiology, School of Dentistiry, Shahid Beheshti University of Medical Sciences, Tehran, Iran; b Department of Oral and Maxillofacial Surgery, School of Dentistiry, Shahid Beheshti University of Medical Sciences, Tehran, Iran

**Keywords:** Aneurysmal bone cysts, Dentigerous cyst, Mandibular diseases, Giant cell granuloma, Cone-beam computed tomography, Panoramic radiography, Histopathology, Differential diagnosis, Jaw cysts

## Abstract

Aneurysmal bone cysts (ABCs) are rare, benign, expansile lesions of the jaws, often associated with odontogenic cysts like dentigerous cysts, presenting diagnostic challenges due to their variable radiographic and histopathological features. This case report describes an ABC associated with a dentigerous cyst in the mandible, initially misdiagnosed as central giant cell granuloma (CGCG). A 19-year-old male presented with an asymptomatic semi-erupted right mandibular third molar (#48), and panoramic radiography revealed a multilocular radiolucent lesion (22.9 × 46.5 × 20.4 mm) in the posterior mandible and ascending ramus, with features including curved and coarse septa, hazy bone pattern, possible root resorption, mild cortical expansion on CBCT, focal perforation, and posterior displacement of the mandibular nerve canal. Initial incisional biopsy suggested CGCG due to multinucleated giant cells and fibrous stroma, but excisional biopsy confirmed an ABC with sinusoidal spaces alongside a dentigerous cyst lined by nonkeratinized stratified squamous epithelium. This case highlights the critical role of CBCT and excisional biopsy in diagnosing ABCs associated with dentigerous cysts, distinguishing them from CGCG and other lesions through integrated clinical, radiographic, and histopathological evaluation.

## Introduction

Aneurysmal bone cysts (ABCs) are benign, expansile osteolytic lesions of the jaws, characterized by blood-filled cystic spaces lined by fibrous tissue and multinucleated giant cells, which may occur as primary lesions or secondary to other pathologies, such as odontogenic cysts or tumors [1]. Although rare in the jaws, ABCs account for approximately 1%-2% of bone lesions in the head and neck region and are often associated with rapid growth, cortical expansion, and occasional perforation, posing diagnostic and therapeutic challenges [2,3]. In this case report, an ABC associated with a dentigerous cyst was identified in the ascending ramus of the mandible, initially misdiagnosed as central giant cell granuloma (CGCG) based on incisional biopsy but confirmed as an ABC with a dentigerous cyst component following excisional biopsy. This case highlights the diagnostic complexity of ABCs due to their variable radiographic and histopathological presentations, particularly when associated with odontogenic cysts.

The differential diagnosis for jaw lesions with multilocular or mixed radiopaque-radiolucent patterns is extensive, including odontogenic cysts (eg, dentigerous cyst, odontogenic keratocyst), odontogenic tumors (eg, ameloblastoma), fibro-osseous lesions (eg, fibrous dysplasia), central giant cell granuloma (CGCG), and systemic conditions like hyperparathyroidism presenting with brown tumors [4,5]. CGCG, a benign intraosseous lesion, is a key differential due to its histopathological similarity to ABC, as both feature multinucleated giant cells and fibrous stroma, often leading to diagnostic confusion [6]. Dentigerous cysts, typically associated with the crowns of unerupted teeth such as mandibular third molars, may rarely develop secondary ABC components, which can enhance the lesion’s aggressive behavior and radiographic complexity [7].

Laboratory investigations, including serum calcium, phosphorus, and parathyroid hormone (PTH) levels, are critical to exclude systemic conditions like hyperparathyroidism, which can mimic ABC or CGCG [5].

The pathogenesis of ABCs remains incompletely understood but is thought to involve vascular disturbances, possibly triggered by trauma, inflammation, or underlying lesions like dentigerous cysts, leading to the formation of blood-filled spaces [8]. In the jaws, ABCs often present as multilocular radiolucencies with wispy or coarse septa, fluid levels, and cortical thinning or perforation, as observed in this case [9]. Cone-beam computed tomography (CBCT) is essential for evaluating ABCs, providing detailed visualization of their internal structure and relationship with adjacent anatomical structures [10]. In this case, CBCT revealed a mixed multilocular lesion with wispy, curved septa and hazy new bone formation, initially suggestive of CGCG but consistent with an ABC associated with a dentigerous cyst [11]. Excisional biopsy was pivotal for definitive diagnosis, as incisional biopsies may not fully capture the hemorrhagic and cystic components characteristic of ABCs [12].

The association of ABC with a dentigerous cyst in this case, located in the ascending ramus, underscores the diagnostic challenges of these lesions, particularly their nonclassic presentation and potential for aggressive behavior, such as cortical perforation [13]. The incidental discovery of this lesion in an asymptomatic patient emphasizes the importance of routine radiographic evaluation for unerupted teeth, which may harbor cystic or neoplastic changes [14]. This case highlights the critical role of integrating clinical, radiographic, and histopathological findings to accurately diagnose ABCs, especially when associated with odontogenic cysts, and to distinguish them from similar lesions like CGCG.

## Case presentation

A 19-year-old male patient presented to the dental clinic for evaluation of a semi-erupted right mandibular third molar (#48), identified during a routine dental examination. The patient reported no pain, tenderness, or facial asymmetry. Clinical examination revealed no palpable mass or mucosal discoloration. Intraoral examination showed normal gingival and mucosal tissues in the posterior right mandible, with no inflammation, ulceration, or mucosal discoloration. The adjacent teeth (#46 and #47) appeared clinically normal with normal mobility, periodontal health, and no caries or occlusal abnormalities, consistent with the asymptomatic presentation. The patient denied any history of trauma, infection, or systemic diseases, and his medical and dental history was unremarkable.

Laboratory investigations, including a complete blood count (CBC), coagulation profile, and biochemical tests, were conducted to assess systemic health. The CBC results were normal: white blood cells 6.1 × 10³/µL (normal: 4-11 × 10³/µL), hemoglobin 13.2 g/dL (normal: 13-17 g/dL), platelets 320 × 10³/µL (normal: 150-400 × 10³/µL), neutrophils 52% (normal: 40-75%), and eosinophils 0.4% (normal: 0%-6%). Coagulation parameters were normal: prothrombin time 11.8 seconds (normal: 11-13.5 seconds) and partial thromboplastin time 26.4 seconds (normal: 25-35 seconds). Biochemical tests showed serum calcium 9.8 mg/dL (normal: 8.6-10.3 mg/dL), urea 24 mg/dL (normal: 10-50 mg/dL), creatinine 0.9 mg/dL (normal: 0.6-1.2 mg/dL), sodium 142 mEq/L (normal: 135-145 mEq/L), and potassium 4.1 mEq/L (normal: 3.5-5.0 mEq/L). C-reactive protein was 0.3 mg/L (normal: <10 mg/L), and serologic tests for hepatitis B, hepatitis C, and HIV were negative. These findings ruled out systemic conditions such as hyperparathyroidism, suggesting a localized pathology.

Initial panoramic radiography (OPG) revealed a semi-erupted right mandibular third molar (#48), vertically positioned, inferiorly and lingually oriented, with a multilocular radiolucent lesion measuring 22.9 × 46.5 × 20.4 mm in the posterior mandible and ascending ramus.

**Fig. 1:**
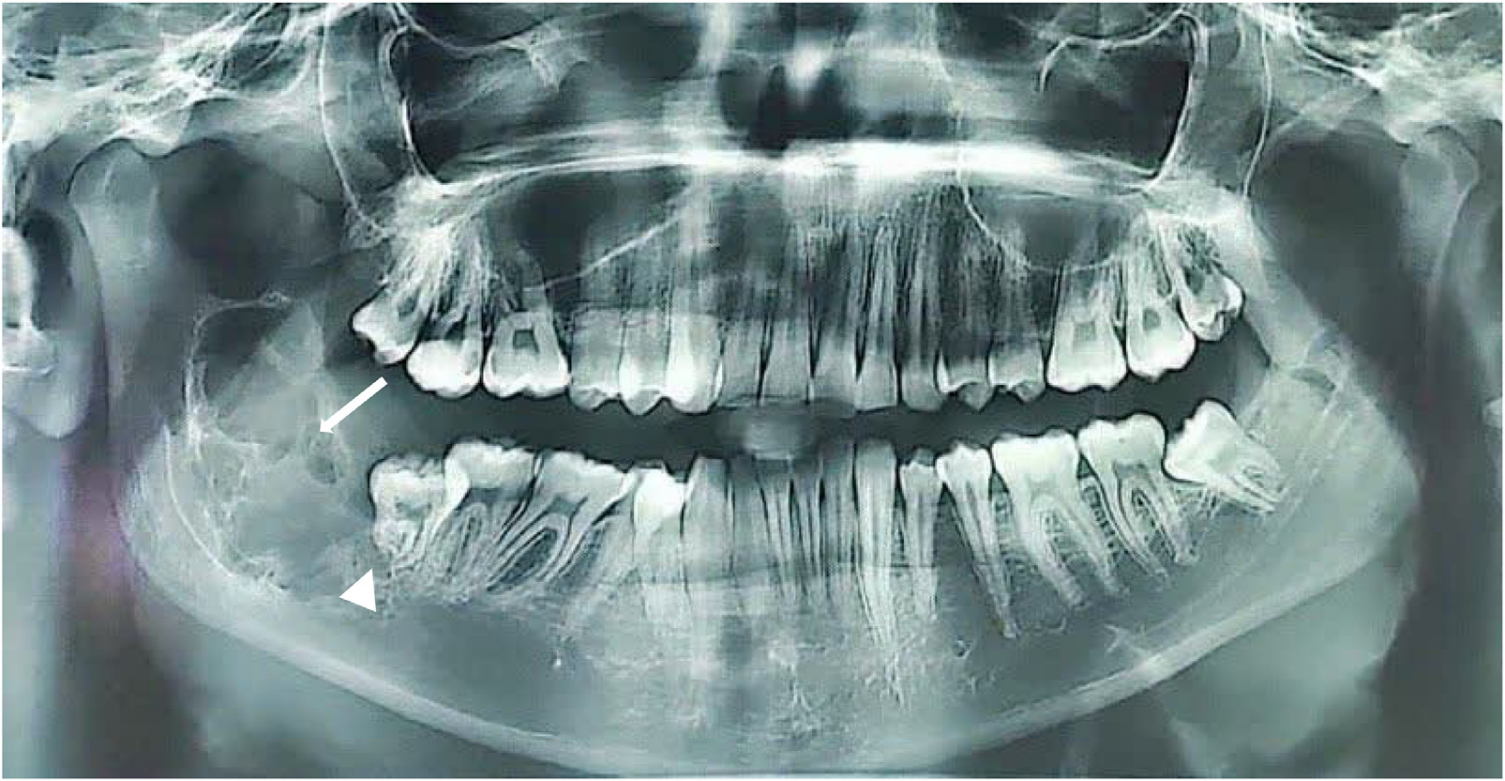
Panoramic radiograph (orthopantomogram, OPG) in a 2D projection plane demonstrating a multilocular radiolucent lesion in the posterior mandible and ascending ramus associated with a semi-erupted right mandibular third molar (#48), which is vertically positioned, inferiorly and lingually oriented. The lesion features curved and coarse septa in addition to typical wispy septa (white arrows), irregular bone destruction with a hazy and blurred pattern, and possible root resorption of tooth #48 (white arrowhead). A normally erupted contralateral third molar (#38) is visible for comparison.

Differential diagnoses included central giant cell granuloma (CGCG), ameloblastoma, and odontogenic keratocyst (OKC). The lesion exhibited curved and coarse septa in addition to usual wispy septa, irregular bone destruction with a hazy and blurred pattern, and possible root resorption of the right mandibular third molar (#48). The contralateral third molar (#38) was normally erupted, highlighting asymmetry (Fig. 1).

**Fig. 2:**
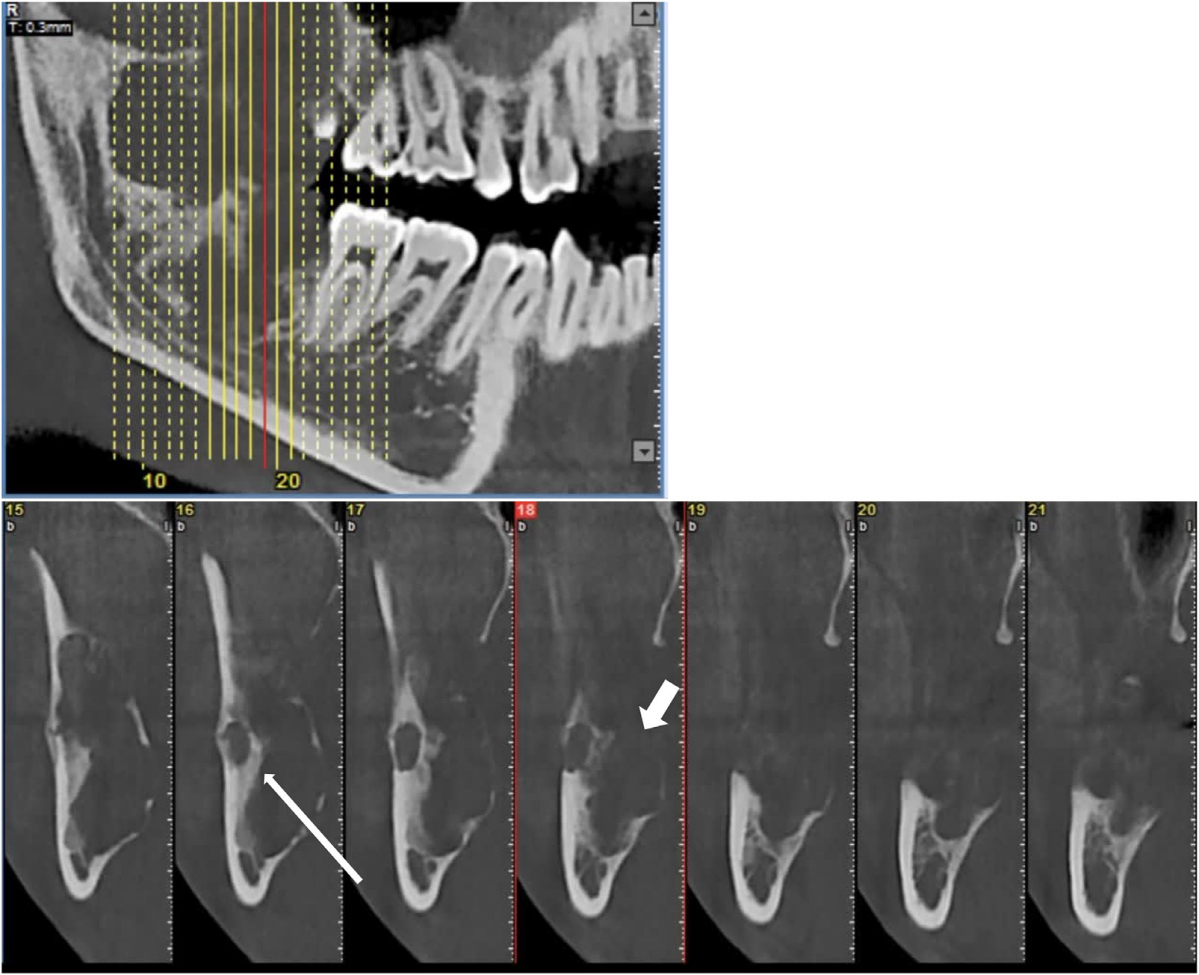
Cone-beam computed tomography (CBCT) images reconstructed in pseudo-panoramic and cross-sectional planes, demonstrating a multilocular radiolucent lesion with curved and coarse septa (white arrows) in addition to the usual wispy septa, a hazy and blurred bone pattern, mild cortical expansion, focal perforation (white thick arrow), and posterior displacement of the mandibular nerve canal without periosteal reaction. These views allow for a detailed assessment of the lesion's depth and multilocular pattern with curved septa.

Cone-beam computed tomography (CBCT) imaging included a pseudo-panoramic reconstruction and cross-sectional view, confirming a multilocular radiolucent lesion of the same size, associated with tooth #48, showing multiple cavities indicative of an aneurysmal bone cyst, along with mild cortical expansion and focal perforation. The pseudo-panoramic image exhibited curved and coarse septa in addition to usual wispy septa, a hazy and blurred pattern, and possible root resorption of tooth #48, while the cross-sectional view delineated the lesion’s depth and multilocular pattern with curved and coarse septa in addition to usual wispy septa, reinforcing the multiple cavities and focal perforation (Fig. 2).

**Fig. 3:**
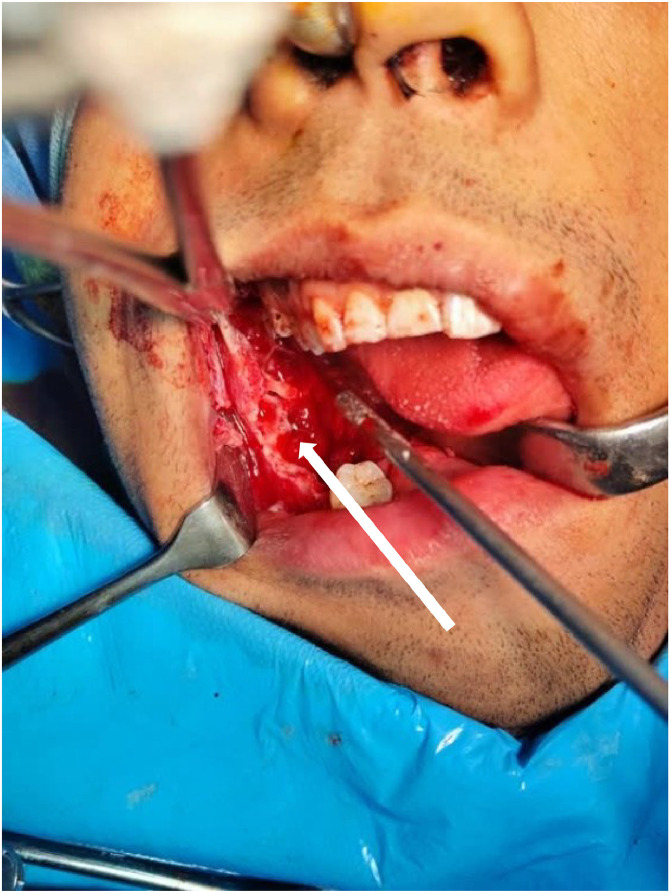
Intraoperative photograph showing the preoperative assessment with no prior intervention, with visible coarse septa (white arrow) and a multilocular appearance consistent with the radiographic diagnosis of a dentigerous cyst with a secondary aneurysmal bone cyst.

During the surgical intervention, preoperative assessment showed no prior intervention, with visible coarse septa and a multilocular appearance consistent with the radiographic diagnosis of a dentigerous cyst with a secondary aneurysmal bone cyst (Fig. 3).

**Fig. 4:**
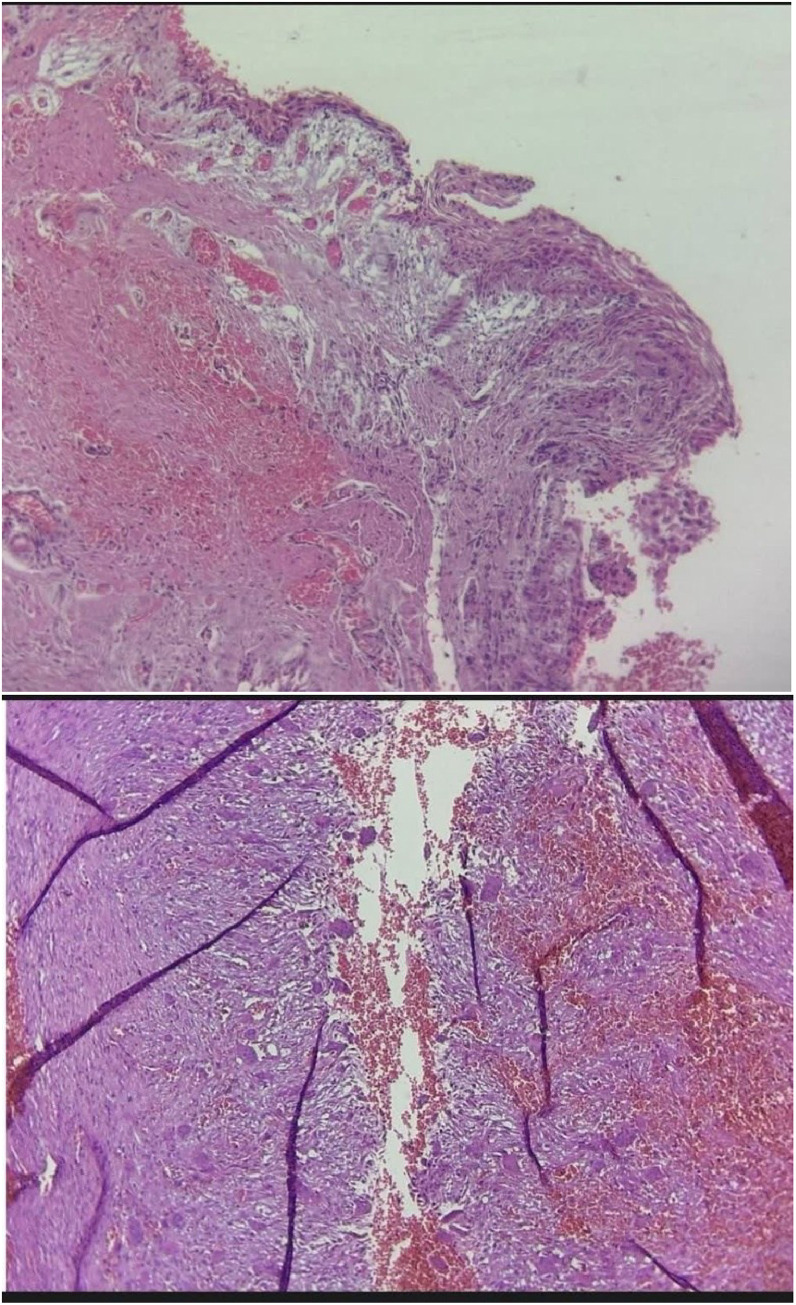
Histopathological slide showing a dentigerous cyst lined by nonkeratinized stratified squamous epithelium, with an adjacent aneurysmal bone cyst exhibiting blood-filled sinusoidal spaces, fibrous septa, and multinucleated giant cells, confirming the final diagnosis.

An incisional biopsy initially suggested a central giant cell granuloma (CGCG) due to the presence of multinucleated giant cells and fibrous stroma. Based on the initial radiographic findings (mentioned before) and histopathological features, the differential diagnoses were refined to include CGCG accompanied by a fibro-osseous lesion and low-grade osteosarcoma, as these conditions better accounted for the aggressive radiographic appearance and histological findings compared to the initial broader differential diagnoses of CGCG, ameloblastoma, and OKC. However, due to the similar appearance to CGCG but the presence of sinusoidal spaces in the excisional biopsy, the diagnosis was established as an aneurysmal bone cyst (ABC). Further histopathological examination and correlation with radiographic findings confirmed a dentigerous cyst lined by nonkeratinized stratified squamous epithelium, with an adjacent aneurysmal bone cyst characterized by blood-filled spaces separated by fibrous septa and multinucleated giant cells (Fig. 4).

**Fig. 5:**
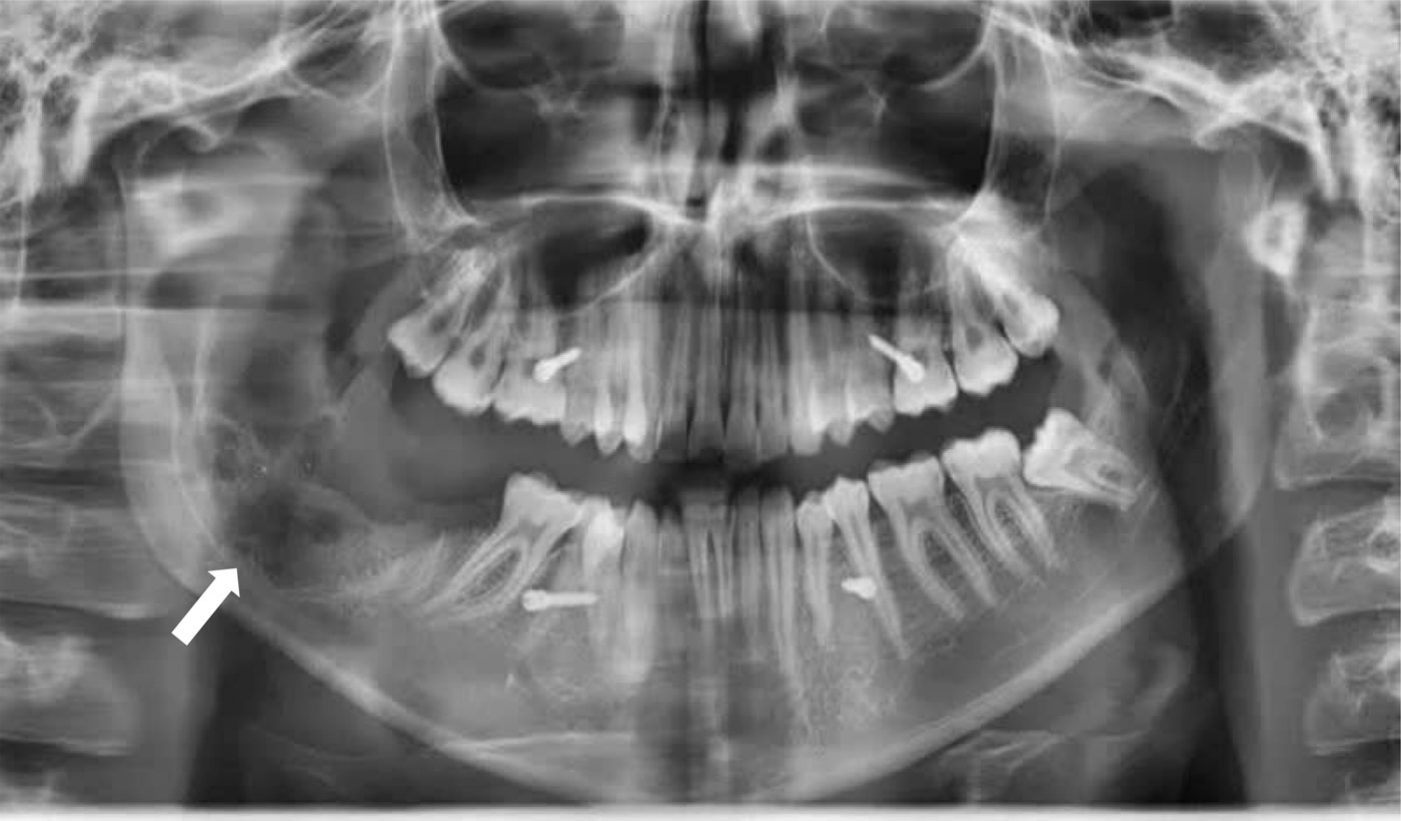
Follow-up panoramic radiograph obtained one month after surgery, showing a significant reduction in the multilocular radiolucency previously associated with the right mandibular third molar (#48), with no further progression of resorption in the adjacent right second molar (#47), indicating a favorable healing response (white arrow).

A follow-up panoramic radiograph obtained one month after surgery demonstrated a significant reduction in the multilocular radiolucency previously associated with the right mandibular third molar (#48), with no further progression of resorption in the adjacent right second molar (#47), indicating a favorable healing response. The patient was referred to an oral and maxillofacial surgeon for further management and follow-up (Fig. 5).

The radiographic presentation of this lesion was critical in refining the differential diagnosis. Panoramic radiography revealed a multilocular radiolucent lesion associated with a semi-erupted mandibular third molar, with irregular bone destruction and possible root resorption, initially suggestive of a dentigerous cyst or CGCG [9]. The absence of periosteal reaction and the lesion’s extension beyond the bone further supported the diagnosis of an ABC, as these features are consistent with its expansile and locally aggressive nature [15]. CBCT’s ability to provide high-resolution, three-dimensional visualization was pivotal in this case, as it allowed for a detailed assessment of the lesion’s internal architecture, cortical integrity, and relationship with the mandibular nerve canal [16]. This is particularly important in the ascending ramus, where proximity to vital structures like the inferior alveolar nerve increases the risk of neurological complications during surgical intervention [17]. Previous studies have highlighted CBCT’s superiority over panoramic radiography in detecting subtle radiographic features, such as fluid levels or septa morphology, which are critical for distinguishing ABCs from CGCG or other jaw lesions [18]. The identification of possible root resorption of the mandibular third molar on CBCT further underscored the lesion’s aggressive behavior, necessitating careful surgical planning [19].

The pathogenesis of ABCs remains incompletely understood but is thought to involve vascular disturbances, possibly triggered by trauma, inflammation, or an underlying lesion such as a dentigerous cyst [15]. In this case, the dentigerous cyst likely served as the primary lesion, with the ABC developing secondarily, a phenomenon reported in approximately 30% of jaw ABCs [20]. The dentigerous cyst, typically associated with the crown of an unerupted tooth, may have initiated a localized vascular event, leading to the formation of blood-filled cystic spaces characteristic of an ABC [21]. The histopathological findings of nonkeratinized stratified squamous epithelium lining the cyst, adjacent to sinusoidal spaces with multinucleated giant cells, support this hypothesis [8]. The lesion’s location in the ascending ramus and its aggressive radiographic features, including cortical perforation, suggest a more complex interplay between the cystic and aneurysmal components, potentially driven by increased vascular pressure or inflammatory mediators [15].

The incidental discovery of this lesion in an asymptomatic patient highlights the importance of routine radiographic evaluation of unerupted or semi-erupted teeth, which may harbor cystic or neoplastic changes [22]. The lesion’s proximity to the mandibular nerve canal, as observed on CBCT, poses significant challenges for surgical management, as resection or enucleation carries a risk of nerve injury and resultant paresthesia [23]. The cortical perforation and possible root resorption further suggest a more aggressive behavior, potentially requiring a comprehensive surgical approach, such as enucleation with curettage or peripheral ostectomy, to minimize recurrence [16]. Adjunctive therapies, such as intralesional steroid injections or sclerotherapy, have been reported for ABCs in some cases, but their efficacy in the jaws remains under investigation [24].

The initial misdiagnosis of CGCG based on incisional biopsy underscores the limitations of limited tissue sampling in distinguishing ABCs from other giant cell-rich lesions [25]. Excisional biopsy was critical in this case, as it allowed for a comprehensive evaluation of the lesion’s hemorrhagic and cystic components, which are often missed in smaller samples [26]. This case also highlights the importance of correlating radiographic and histopathological findings, as the CBCT’s depiction of wispy septa was more suggestive of an ABC than the initial CGCG diagnosis [14].

## Discussion

Aneurysmal bone cysts (ABCs) are rare, benign, expansile lesions of the jaws, characterized by blood-filled cystic spaces lined by fibrous septa and multinucleated giant cells. This case report describes an ABC associated with a dentigerous cyst in the ascending ramus of a mandible in a 19-year-old male, initially misdiagnosed as central giant cell granuloma (CGCG) based on incisional biopsy. The definitive diagnosis was established through excisional biopsy, which revealed the characteristic sinusoidal spaces of an ABC alongside a dentigerous cyst component, highlighting the diagnostic complexity of such lesions. This case underscores the importance of integrating clinical, radiographic, and histopathological findings to differentiate ABCs from similar lesions, particularly CGCG, and emphasizes the critical role of cone-beam computed tomography (CBCT) in evaluating nonclassic jaw lesions.

The lesion’s presentation in the ascending ramus, an uncommon location for both ABCs and dentigerous cysts, posed significant diagnostic challenges. ABCs typically occur in long bones and are rare in the jaws, accounting for only 1%-2% of head and neck bone lesions [27]. When occurring in the jaws, ABCs are often associated with underlying pathologies such as dentigerous cysts, as seen in this case, or other odontogenic lesions [2]. Similar associations have been previously reported, such as an aneurysmal bone cyst with a dentigerous cyst in the maxillary sinus, contrasting with the mandibular location in the present case [28].

The initial incisional biopsy suggested CGCG due to the presence of multinucleated giant cells and fibrous stroma, a common histopathological overlap with ABCs [6]. Both lesions share similar microscopic features, including giant cells and fibrous tissue, which can lead to misdiagnosis, particularly when sampling is limited, as in incisional biopsies [4]. The excisional biopsy, however, revealed blood-filled sinusoidal spaces, a hallmark of ABCs, alongside a dentigerous cyst lined by nonkeratinized stratified squamous epithelium, confirming the diagnosis [7].

The differential diagnosis for this multilocular radiolucent lesion with wispy, curved septa and cortical perforation included CGCG, ameloblastoma, odontogenic keratocyst (OKC), fibro-osseous lesions (eg, fibrous dysplasia), and low-grade osteosarcoma [29]. Ameloblastoma was considered due to its multilocular radiographic appearance and association with unerupted teeth, but its histopathological features, such as epithelial proliferation, were absent [10]. OKC was another possibility given its association with impacted teeth, but the absence of keratinized epithelium and the presence of blood-filled spaces ruled it out [30]. Fibro-osseous lesions like fibrous dysplasia were less likely due to the lesion’s aggressive behavior, including cortical perforation and lack of characteristic ground-glass radiopacity [31]. Low-grade osteosarcoma was considered due to the lesion’s invasive behavior and hazy bone formation, but the absence of malignant cellular features on histopathology excluded this diagnosis [32]. Systemic conditions like hyperparathyroidism, which can present with brown tumors mimicking ABC or CGCG, were ruled out by normal serum calcium (9.8 mg/dL), phosphorus, and parathyroid hormone levels [33].

## Conclusion

This case of a dentigerous cyst associated with an aneurysmal bone cyst (ABC) in the mandible of a 19-year-old male illustrates the diagnostic intricacies of multilocular jaw lesions. Initially mistaken for a central giant cell granuloma (CGCG), ameloblastoma, or odontogenic keratocyst due to its multilocular radiolucency and wispy septa on panoramic radiography, the lesion’s true nature was elucidated through advanced imaging and comprehensive histopathology. Cone-beam computed tomography (CBCT) revealed critical features, including cortical perforation and posterior displacement of the mandibular nerve canal, guiding the refined differential diagnoses of CGCG with a fibro-osseous lesion and low-grade osteosarcoma. The excisional biopsy, identifying sinusoidal spaces alongside a dentigerous cyst, was pivotal in confirming the ABC diagnosis, overcoming the limitations of the initial incisional biopsy’s CGCG suggestion. This case underscores the necessity of routine radiographic screening for unerupted teeth, the superior diagnostic value of CBCT for complex lesions, and the importance of thorough histopathological evaluation to distinguish ABCs from similar entities. Referred for surgical management, the patient’s case highlights the need for meticulous planning to mitigate risks associated with the lesion’s aggressive behavior and proximity to vital structures.

## Patient consent

The patient provided informed consent for his clinical information and images to be included in this case report. He understood the purpose of the publication, how his privacy would be protected, and agreed voluntarily to share his data for research purposes.
